# Big data from a popular app reveals that fishing creates superhighways for aquatic invaders

**DOI:** 10.1093/pnasnexus/pgac075

**Published:** 2022-06-08

**Authors:** Jessica L Weir, Kirsten Vacura, Jay Bagga, Adam Berland, Kieran Hyder, Christian Skov, Johan Attby, Paul A Venturelli

**Affiliations:** Department of Biology, Ball State University, Muncie 47306, IN, USA; Department of Biology, Ball State University, Muncie 47306, IN, USA; Department of Computer Science, Ball State University, Muncie, IN 47306, USA; Department of Geography, Ball State University, Muncie, IN 47306, USA; Center for Environment, Fisheries and Aquaculture Science (Cefas), Lowestoft, Suffolk NR33 0HT, UK; School of Environmental Sciences, University of East Anglia, Norwich Research Park, Norwich, Norfolk NR4 7TJ, UK; National Institute of Aquatic Resources, Technical University of Denmark, Silkeborg 8600, Denmark; Fishbrain, 118 27 Stockholm, Sweden; Department of Biology, Ball State University, Muncie 47306, IN, USA

**Keywords:** invasive species, human dimensions, network, big data

## Abstract

Human activities are the leading cause of biological invasions that cause ecologic and economic damage around the world. Aquatic invasive species (AIS) are often spread by recreational anglers who visit two or more bodies of water within a short time frame. Movement data from anglers are, therefore, critical to predicting, preventing, and monitoring the spread of AIS. However, the lack of broad-scale movement data has restricted efforts to large and popular lakes or small geographic extents. Here, we show that recreational fishing apps are an abundant, convenient, and relatively comprehensive source of “big” movement data across the contiguous United States. Our analyses revealed a dense network of angler movements that was dramatically more interconnected and extensive than the network that is formed naturally by rivers and streams. Short-distanced movements by anglers combined to form invasion superhighways that spanned the contiguous United States. We also identified possible invasion fronts and invaded hub lakes that may be superspreaders for two relatively common aquatic invaders. Our results provide unique insight into the national network through which AIS may be spread, increase opportunities for interjurisdictional coordination that is essential to addressing the problem of AIS, and highlight the important role that anglers can play in providing accurate data and preventing invasions. The advantages of mobile devices as both sources of data and a means of engaging the public in their shared responsibility to prevent invasions are probably general to all forms of tourism and recreation that contribute to the spread of invasive species.

Significance Statement Information about the human networks through which aquatic invasive species are spreading is limited by a lack of extensive human movement data. Big data from a popular fishing app have revealed a dense network of short-distanced movements that combine to form invasion superhighways spanning the contiguous United States. These data also revealed potential invasion fronts and invaded hubs that may be superspreaders for two relatively common aquatic invaders. Our results provide insight into a national network of aquatic invasions, and highlight both the potential of big data sources, and the role that anglers can play in preventing aquatic invasions.

## Introduction

The growth and integration of human transportation networks has facilitated the spread of invasive species (1) that disrupt ecosystem services (2), reduce the diversity and abundance of native species (3), threaten endangered species (4), and trigger trophic cascades (2, 5). These ecological impacts have socio-economic consequences for food security, industry, and human health (6, 7) that total more than US$26 billion globally each year (8).

Human movement data for tracking the spread of invasive species are difficult to collect. Conventional approaches include travel diaries, interviews, and surveys (9–16) that can be biased in myriad ways (17, 18), and tend to have high costs and/or low response rates that limit data in space and time (19, 20). Mobile smart devices can be an efficient source of quality, high-resolution, and individualized movement data over broad spatial scales (21, 22). A total of 85% of adults in the United States use smartphones (23) that collect geolocation data. Spatiotemporal data from mobile smart devices and social software applications (apps) have been used to predict the spread of COVID-19 (24) and other diseases (25), discern human migration patterns (26), track species distributions (27–29), and estimate recreational demand for natural spaces (30, 31). Recent studies suggest that these data can also be used to identify the pathways along which humans spread invasive species (18, 32).

Here, we use 10 years of movement data from the popular fishing app Fishbrain to show how recreational anglers connect > 100,000 lakes across the contiguous United States, and how the resultant connectivity network provides unique insight into the current and future distribution of aquatic invasive species (AIS). Anglers are an important vector for AIS dispersal, but studies have focused on their movement at local or regional scales—often only for large or popular lakes (9, 15, 33). Two notable exceptions used movement data from apps to reveal seasonal connectivity networks among hundreds of lakes in Alberta, Canada (32), and to characterize fishing trips among ∼20,000 lakes in the United States (18). These studies promote the exciting possibility of using mobile data to identify human-mediated pathways of dispersal among a much larger and more diverse subset of lakes at regional to continental scales.

Our study exemplifies how extensive, high-resolution spatiotemporal mobile data can provide an unprecedented glimpse into dispersal patterns and invasion dynamics that ultimately benefit invasive species research and management. We began by contrasting the connectivity of waterbodies in the United States through two common AIS dispersal pathways, including the novel connections that are created by angler movement and the hydrologic connections that occur naturally. We then show how the local movement of anglers combines and spans the continent to form pathways that overlap with the US interstate highway system. We assess the proximity of invaded lakes to this invasion superhighway and identify possible superspreaders for two common and widespread invaders of the United States. We then map areas of the United States that are presumably at high-risk of invasion because of the connections that originate at an invaded lake and terminate at an uninvaded lake.

## Results

We assigned 3,905,675 of the 4,898,603 (78%) freshwater catches that were logged in the United States to lake polygons from the National Hydrography Dataset (NHD). From these catches, sequential trips within a 99-day period resulted in 1,130,241 individual connections by 248,497 anglers. These data described an angler network that comprised 130,498 nodes (lakes) that were connected by 659,669 edges. The largest connected component in the angler network included 95% of lakes, spanned the contiguous United States, and was easy to traverse via shortcuts and redundant pathways (Table 1). In contrast, hydrologic connections formed many small, isolated components rather than a large, interconnected network (Table 1). Only 50,202 (38%) lakes in the angler network were connected by 47,494 edges that represented the downstream flow of river and stream segments. The remaining 80,296 (62%) lakes that comprised the angler network were hydrologically isolated from each other.

**Table 1.** Network terms and values that describe the shape and structure of the angler and hydrologic networks, including their relevance and interpretation to this study.

Term	Definition	Value		Relevance and Interpretation
		Hydrologic	Angler	
Node lake	The fundamental units of a network that are connected by edges and represented as the mid-point of a lake.	50,202	130,498	Lakes were represented by nodes in the network that was connected by both anglers and streamflow. The angler network was composed of more connected lakes than the hydrologic network.
Edge connection	The connections between the nodes and represented as a straight-line path between the mid-points of two connected lakes.	47,494	659,669	Angler movement connected lakes by an order of magnitude more than did rivers and streams.
Component	A group of nodes that are connected to each other by a *path* (a route across a network that runs along edges). Here, we present the number of components and the percent of nodes in the largest component.	4,717	3,022	More components indicated a fragmented network and a lower chance for the spread of AIS. Lakes were more evenly distributed among hydrologic components that reflected river basins, but anglers connected lakes across great distances and in one single large component.
		3%	95%	
Diameter	The longest of all shortest paths measured for the largest component of the network.	31.25	33.42	The diameter for the largest component of the angler and hydrologic network were approximately equal (but the angler network was much larger and more connected, and therefore, much more traversable per lake node).
Density	A value from 0 to 1 that measures the relative number of all edges to all possible edges that could exist for a set of nodes.	1.88 × 10^−5^	3.87 × 10^−5^	A higher density network facilitates dispersal of AIS. The angler network was twice as dense as the hydrologic network with more shortcuts and redundant pathways that connect lakes more fully.
Reciprocity	The number of edges that have two-way flow.	0%	43%	More two-way flow in the network leads to a higher chance of spreading AIS between lakes. The hydrologic network facilitated passive dispersal in the downstream direction, whereas the angler network facilitated dispersal in both directions.

Anglers reinforced 8,905 (18.75%) existing streamflow connections and created reciprocal upstream flow (i.e. in the upstream direction) for 18.8% (8,931) of hydrologic connections. Anglers created 650,764 connections between lakes that were not already connected by rivers or streams.

**Fig. 1.**
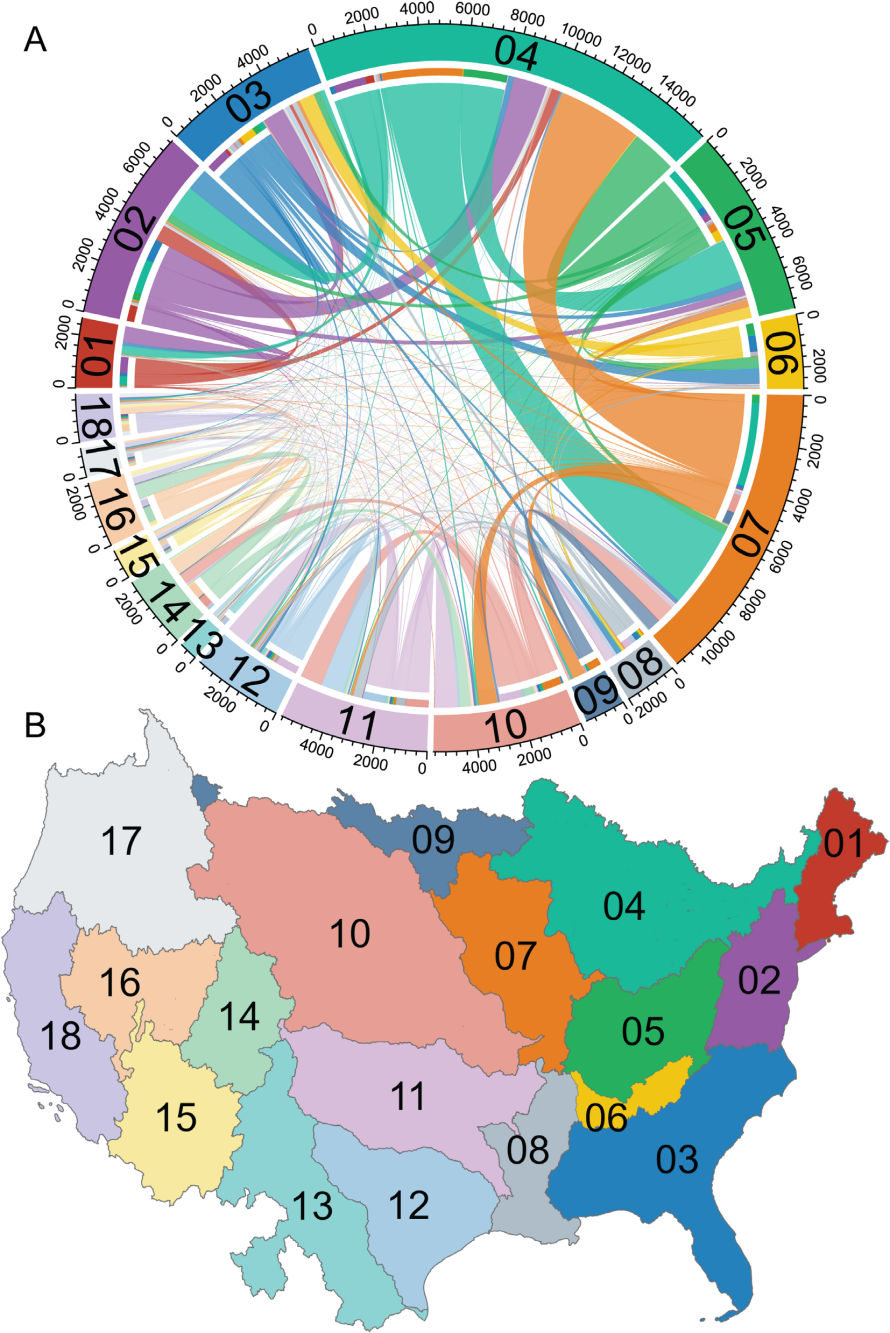
The 18 major river basins of the contiguous United States are connected by anglers across natural hydrologic boundaries. (A) A map of the 18 major river basins in the contiguous United States that extend into parts of Canada and Mexico. These basins exist because waterbodies are separated by natural hydrologic boundaries, except at the basin outlet. The * represents an area where network lakes are hydrologically connected across the river basin boundary. (B) A chord diagram showing the connections from angler movement among the 18 major river basins. The outer ring and color correspond to the region in which the angler connection originated, and the inner ring corresponds to the basin in which those connections terminate. Thicker lines indicate more movement, and therefore, higher propagule pressure. Basins: 01 New England, 02 Mid Atlantic, 03 South Atlantic–Gulf, 04 Great Lakes, 05 Ohio, 06 Tennessee, 07 Upper Mississippi, 08 Lower Mississippi, 09 Souris–Red–Rainy, 10 Missouri, 11 Arkansas–White–Red, 12 Texas–Gulf, 13 Rio Grande, 14 Upper Colorado, 15 Lower Colorado, 16 Great Basin, 17 Pacific Northwest, and 18 California.

Major river basins within the contiguous United States were hydrologically isolated from each other by definition (except for terminal outlets; Figure 1A), but highly connected by angler movement (Figure 1B). Each major basin was most connected by anglers to the major basins that were adjacent to it, and the strongest interbasin angler connections were between the Great Lakes and Upper Mississippi basins (Figure 1B). The strongest connection between two nonadjacent basins originated in the South Atlantic Gulf basin and terminated in the Great Lakes basin. Angler connections were strongest among basins in the eastern United States, regardless of basin size or lake number. Only one major river basin pair was not connected by angler movement: from the Rio Grande to the Souris–Red–Rainy basin.

**Fig. 2.**
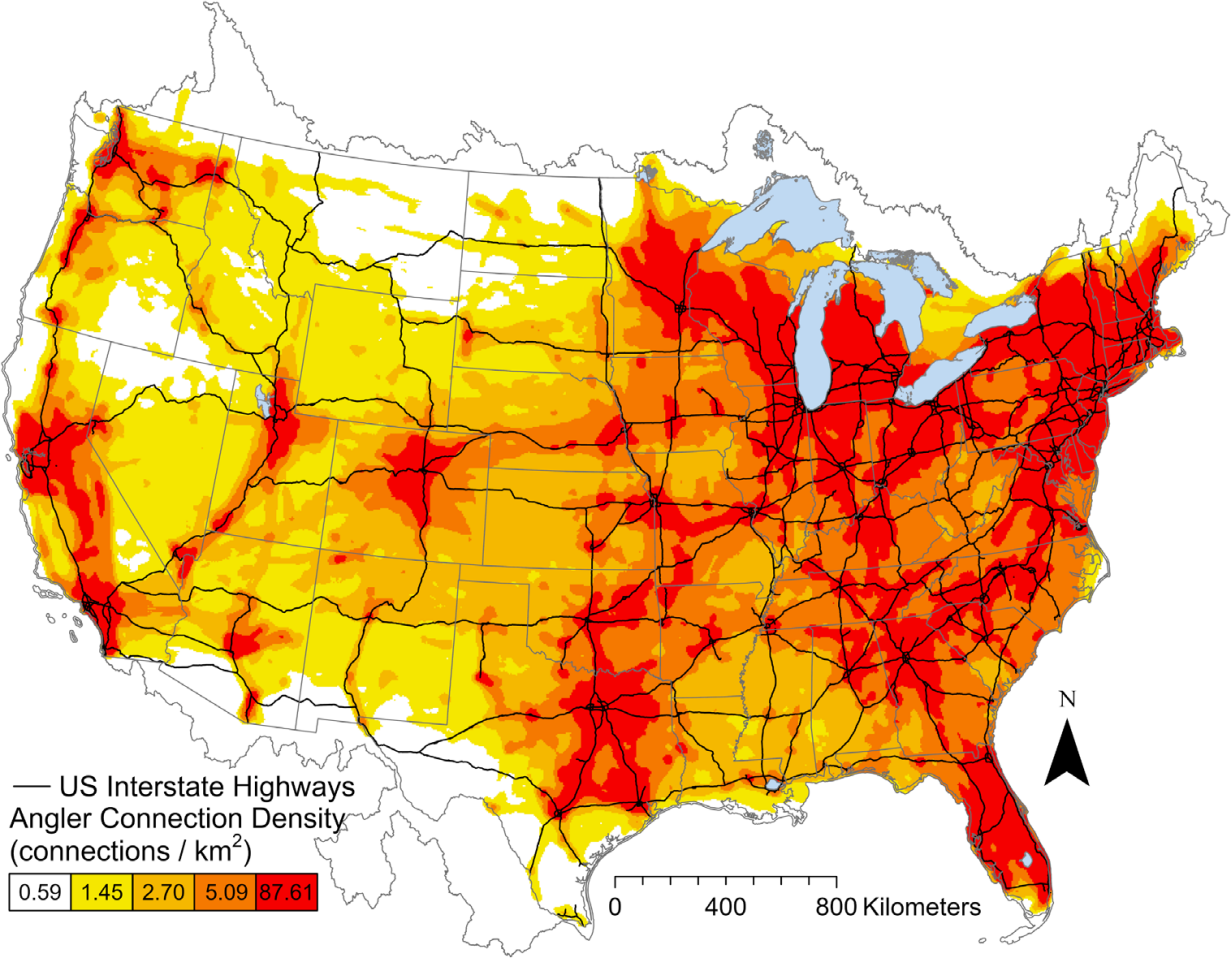
Angler connection density (edges per km^2^) across all 18 major river basins of the contiguous United States and parts of Canada and Mexico. Black lines represent the interstate highway system of the United States. The numbers on the colored scale bar represent the upper value for angler connection density quintiles.

**Fig. 3.**
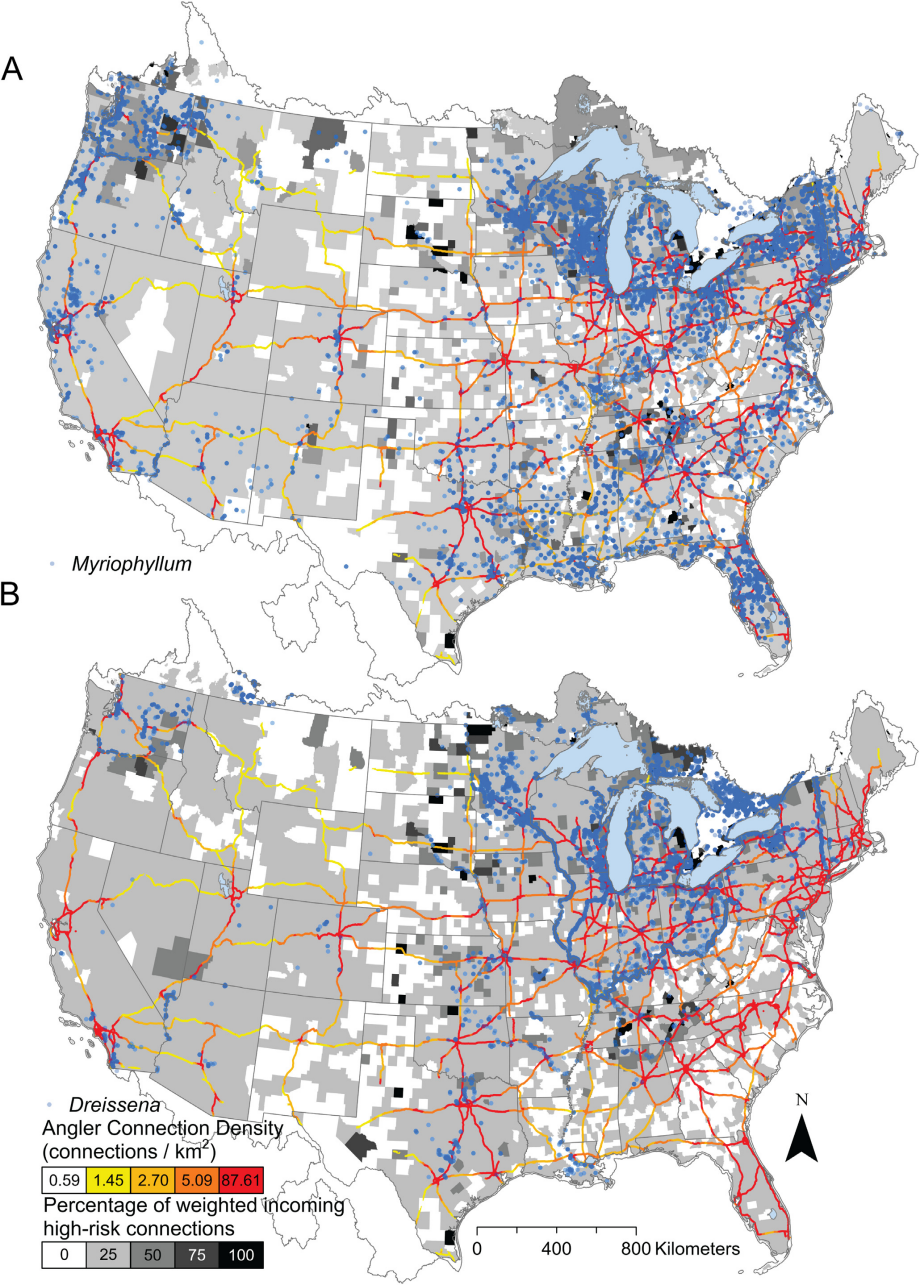
The invasion superhighway combining invasion pathways along major highways in North America. Maps include the point distribution of invasive (A) *Myriophyllum* species and (B) *Dreissena* species. Overlapping points appear with greater color intensity. Counties (United States) and census units (Canada) are in grayscale to represent the percentage of weighted, incoming connections that are presumably at high-risk because they originated from an invaded lake (by one or both *Dreissena* species and *Myriophyllum* species) and terminated at an uninvaded lake.

**Fig. 4.**
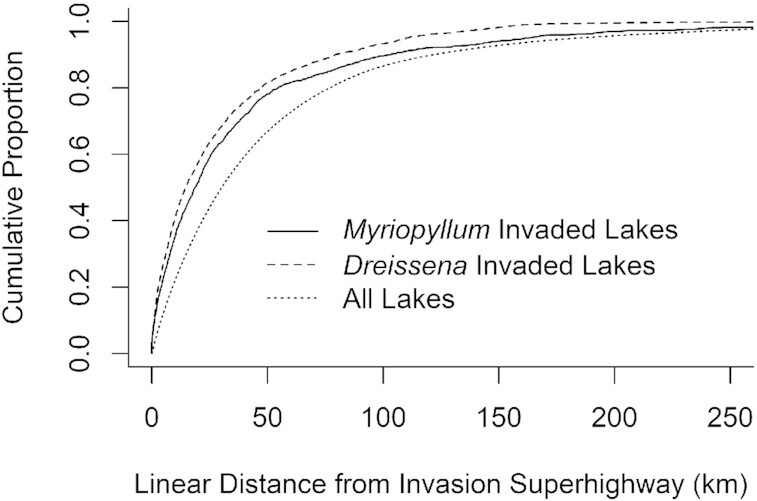
The cumulative proportion of all lakes > 0.06 ha, lakes invaded by *Myriophyllum*, and lakes invaded by *Dreissena* with increasing distance (km) from the invasion superhighway. Distances occurred at up to 537 km (not shown).

Half of all angler movements occurred ≤ 7 days apart (Figure S1, Supplementary Material) and were between lakes that were separated by ≤ 20 linear km (Figure S2, Supplementary Material). These frequent, short-distance movements combined to form a continuous network that connected lakes across the contiguous United States (Figure 2). These areas of high connection density overlapped with the interstate highway network in the United States (Figure 2). Average edge connection density was highest at < 20 km from the US interstate system with an average of 7.1 connections/km^2^ and decreased with greater distance from the interstate. The average connection density at nonoverlapping bands at 40, 60, 80, and 100 km from the interstate highway was 4.3, 3.1, 2.4, and 1.9 connections/km^2^, respectively. Parts of the interstate highway system that overlapped with the highest density of angler connectivity created the invasion superhighway (Figure 3). Lakes invaded by *Myriophyllum* (mean = 29.7 km) and *Dreissena* (mean = 40.2 km) species were closer to the invasion superhighway than all lakes (51.4 km; t(3298.5) = 32.477, *P* < 0.001; t(1673.1) = 7.4, *P* < 0.001). Half of all lakes that contained invasive milfoil or mussels were within ∼19 linear km of this “invasion highway” (Figure 4). Approximately 13% of movement was from invaded to uninvaded lakes, but this value varied spatially (Figure 3). High-risk connections, defined as angler connections from an invaded lake to an uninvaded lake, were rare in regions with few lakes and/or AIS (e.g. the center of the continent), and in regions with many lakes that contained AIS (e.g. central Florida in the case of milfoil). High-risk connections involving both milfoil and mussels were common along potential invasion fronts that spanned the center of the continent and the edge of the Appalachian Mountains in the Southeast (Figure 3). High-risk connections involving milfoil were also common in the Northwest (Figure 3A).

## Discussion

Our study is the first to reveal that movements by anglers—particularly over short-distances—combine to form an “invasion superhighway” that spans the contiguous United States, is fundamentally different from the natural network of rivers and streams, and provides important insight into the current and potential future distribution of AIS. Our results are consistent with local and regional studies that used survey, interview, and inspection data to reveal highly connected networks that are dominated by movements over short distances and timeframes, cross natural and political boundaries, and overlap with transportation networks and/or the distribution of AIS (e.g. (9–16, 33–36). However, our study used passive data from 2 to 3 orders of magnitude more lakes to reveal this network at an unprecedented spatial scale and spatiotemporal resolution. Our work is also consistent with and complements national analyses that identified overlapping hotspots of fishing demand and AIS diversity (e.g. the Great Lakes) (37) and showed that most anglers connect lakes over short distances and timeframes (18).

Detailed movement data from throughout the contiguous United States are critical to predicting and preventing the spread of AIS. For example, identifying lakes that are either superspreaders (highly connected and infected) or highly vulnerable (highly connected but uninfected) can help to prioritize resources for surveillance, education, control, and enforcement (38). Connection weight and direction are also proxies for propagule pressure and colonization rate, which can be combined with hydrologic connectivity (39), establishment probability (40–42), and projected impacts (43) to assess risk, and optimize prevention and control across a landscape (44, 45). Whereas these approaches have traditionally been limited by a paucity of movement data, apps that track user location appear to be an abundant, cost-effective, and relatively comprehensive data source for any area of interest. Indeed, we were able to identify the lakes that are likely to be the largest superspreaders and most vulnerable in all 48 states of the contiguous United States (Table S1, Supplementary Material) and identify possible species-specific invasion fronts. Data of this nature enable interjurisdictional coordination that is essential to AIS control (45), but coordination can be difficult to achieve with conventional data. App-based movement data create opportunities for exciting avenues of AIS research. For example, the presence of invasion fronts in regions that have many infected lakes and high fishing effort (e.g. the Great Lakes) is an opportunity to identify lake characteristics that contribute to resiliency to AIS. Scaling studies to a river basin could promote further exploration of the variation of activity and connectivity that occurs across the United States. Angler app data also include activities that may be important to AIS dispersal but are rarely captured by traditional methods—for example, fishing from nonmotorized boats or from shore—and connections involving small and/or private lakes and ponds (46, 47). Angler app data include catches from rivers and streams but assigning these catches to linear features that are to be treated as nodes within a network framework is a challenge that requires further study. Other types of online digital data, like photos from social media and search volumes (48, 49), may provide insight into the characteristics that make sites attractive to visitors, and thereby influence the spread of AIS.

Research is also needed to characterize movement dynamics over time (e.g. seasonal and annual trends) (32) and by angler type (e.g. avid vs. occasional) (50). Finally, although crowd-sourced, social media data appear to be a reliable proxy for lake visits (30, 51) and our results are qualitatively consistent with local and regional studies (9–16, 33–36), we encourage comparative research to evaluate, calibrate, and validate user-generated movement data relative to both traditional (e.g. survey) (32, 52–54) and emerging (e.g. cell phone location data) (55, 56) approaches. Of particular importance is the extent to which both active and passive location data from app users are representative of the overall population (18).

Our analysis of Fishbrain data also highlights the important role of anglers in generating accurate data and preventing the spread of AIS. Whether or not anglers transport AIS between lakes depends, not only on AIS type, number, and life stage, but also on mitigating behaviors (e.g. cleaning or treating equipment, trip location and frequency) (46). The presence of an invasion superhighway that spans the contiguous United States suggests that angler awareness, education, and behavior remain widespread issues (15, 57, 58). Our interactive map for viewing the spatial details of our results [https://doi.org/10.5281/zenodo.6388121] can stimulate AIS research and control, but also serve as a valuable tool for angler education and outreach. Apps like Fishbrain also offer unique opportunities to engage directly with anglers to emphasize their shared responsibility in preventing the spread of AIS. The advantages of mobile devices as both sources of data and a means of engaging with stakeholders are probably general to all forms of tourism and recreation that contribute to the spread of invasive species (16, 59).

## Materials and Methods

### Study area

Our study focused on the 18 major river basins (i.e. level-2 hydrologic units; Figure 1) that make up the contiguous United States and extend into parts of Canada and Mexico. The hydrography of these basins are described by the US Geological Survey's (USGS) NHD (60), which we downloaded at the highest resolution available (1:24,000). Our network analysis utilized the watershed boundary, flowline, and waterbody layers.

### Network development and comparison

#### The angler network

We obtained time, date, and geographic location data of catch records for freshwater and unknown species reported by recreational anglers between 2011 January 1 and 2021 June 14 using Fishbrain, a smartphone app that is popular among anglers. Users reported an exact catch location via a pin icon that defaulted to user location at the time of logging, and/or an approximate catch location that was based on the coordinates of the waterbody that was closest to the exact location. Users could override the approximate location by searching for the waterbody's name in the app's database. We prioritized exact locations over approximate locations in our analysis because they were more precise and made errors easier to detect.

The exact coordinate location of each catch was plotted using ArcGIS® Pro Version 2.8.2 (ESRI, Redlands, CA), and then assigned to an NHD waterbody (the fundamental unit in our network) if it occurred inside of the polygon boundary, or within 50 m of the boundary. If the exact location coordinates could not be assigned to a waterbody, then the set of coordinates for the approximate location was plotted and assigned using the same methods. We restricted our analysis to fishable lakes, defined as NHD waterbodies that were classified as a lake, pond, or reservoir with a surface area > 0.06 ha (half the size of an Olympic swimming pool). We excluded catches that could not be reliably assigned to a discrete waterbody feature like a lake. This excluded catches from linear features, such as rivers and streams.

The connections in our angler network were created by the movement of individual users between two lakes. Each lake in the resulting connectivity network was represented by a node, and lakes visited by the same angler were connected by an edge (see terms and definitions in Table 1). Each connection was composed of a direction (Lake A to Lake B or vice versa) and a weight (*w*) that decreased from 1 to 0 with the number of days between lake visits (*d*) according to the log–logistic curve (Figure S3, Supplementary Material):

\begin{equation*}
w = \left\lbrace \begin{array}{c} {\frac{{1.023}}{{1 + {{(\frac{d}{{5.341}})}^{1.031}}}},\ d < 99}\\ {0,\ d \ge 99} \end{array}.\right.
\end{equation*}

We derived this function from the results of a literature review of the number of days that various life stages of different invasive plants, invertebrates, and fishes are likely to survive transport by an angler (e.g. in a live well, bait bucket, or attached to a boat or trailer) regardless of temperature (Table S2, Supplementary Material). The resultant decline in the proportion of invasive life stages that we can expect to survive over time is a reasonable proxy for the time-dependent risk of transferring an invasive species. We then summed weights across all directed movements for each pair of lakes. Each connection therefore estimated propagule pressure (the cumulative sum of time-weighted movements) in either direction. Data were unavailable to further weight according to high-risk activities such as fishing with live instead of artificial bait (61).

Each edge was represented by a straight-line path between the midpoints of two lakes that are connected by angler movement. Connection distance was the length of each edge in km. We removed connections that suggested same day travel with a distance > 250 km, next day travel > 500 km, and 2-day travel > 1,500 km to mitigate errors in user reported location or time. Although same-day, long-distance travel by air is possible, this mode of transportation typically involves a small amount of dry gear, and is, therefore, unlikely to be a major vector of AIS (but see (62) regarding floatplanes). We removed connections for two lakes sharing the same name due to errors that were created when a user selected the wrong lake among search results.

#### The hydrologic network

We used the physical stream connections (flowlines) in the NHD to develop a weighted and directed network of hydrologic connectivity for the same set of lakes that comprised the angler network. Edge connections included streams, rivers, or culverts under a roadway. We ignored flow in ditches, small canals, karst systems, pipelines, and underground conduits because these features are nonpermanent, subterranean, impeded by grates, and/or highly artificial, and therefore unlikely to contribute significantly to the passage of AIS (63). We merged any two or more clustered lakes that were separated by < 40 m of permanent stream into a single node for the network analysis. Although we developed the merge rule to rejoin lakes that were bisected by roadways (e.g. Lake Minnetonka; 44.9443°, −93.5797°), we also applied it to natural streams (e.g. 48.0780°, −91.7863°) and lakes that were separated by narrows (e.g. 48.0414°, −91.6990°) because they are ineffective barriers to the passive or autonomous movement of AIS (64). Lakes that were merged in this step were then combined in the angler network, which removed any angler connections between the clustered lakes and moved the midpoint for distance measures to the largest lake polygon.

We identified all first-order, downstream connections between lakes, and weighted these connections according to the USGS classification-based estimate of the proportion of the year that a stream is flowing: 1.00 for perennial and unclassified streams, 0.75 for intermittent streams that flow for most weeks or months, and 0.10 for ephemeral streams that flow for hours or days in response to local rainfall. This approach assumes that the dispersal of AIS is predominantly downstream. Some aquatic organisms can disperse upstream (64), but this ability is limited to certain taxa (65), and impeded by natural and physical barriers (63).

We used the igraph package in R version 3.6.2 to calculate global network statistics for both networks including number of nodes, number of edges, % of isolates, number of components, component size, diameter, density, and reciprocity (66). We also calculated connectivity measures for each node and identified hubs. See Table 1 for a definition of each measure and its relevance to the spread of AIS.

### Invasion superhighway and the known distribution of AIS

By visualizing the density of angler connections in our network, we identified pathways along which AIS are likely to be spread through recreational fishing. We used the line density tool in ArcGIS Pro to calculate a raster layer with values corresponding to the density of edges within a 20-km search radius of each 5 km cell. We used the weight of connections to determine the number of times the line should be counted. We excluded areas in the lowest 20% of density values to highlight the most important invasion pathways. US interstate highways (67) were overlaid with the corresponding densities from the map to form the invasion superhighways. We tested whether the density of angler movement was higher near the US interstate highway system by evaluating the mean density of angler movement in nonoverlapping polygon bands around the interstate highway that terminated at 20, 40, 60, 80, and 100 km from the interstate highway.

We evaluated the known distribution of invasive *Myriophyllum* and *Dreissena* species relative to the invasion superhighway. We compiled coordinate point location of vouchered specimens and reported sightings of these species from the USGS Nonindigenous Aquatic Species Program (NAS) the Early Detection and Distribution Mapping System (EDDMapS), and the USGS Biodiversity Information Serving Our Nation (BISON) (68–70). We combined all exotic *Dreissena* species to include zebra mussels (*D. polymorpha*) and quagga mussels (*D. rostriformis bugensis*), and all exotic *Myriophyllum* species to include Eurasian watermilfoil (*M. spicatum*), parrot feather (*M. aquaticum*), and hybrid milfoil (*M. spicatum* × *M. sibiricum*). An NHD lake was considered as invaded by one or both species if a coordinate point occurred inside or within 50 m of the lake boundary. We used the geodesic distance in the Near tool in ArcGIS Pro to calculate a straight-line distance from every invaded lake to the invasion superhighway and determined the proportion of invaded lakes near to the invasion superhighway. We tested whether invaded lakes were closer to the invasion superhighway compared to all lakes > 0.06 ha by comparing the mean distance of invaded lakes and all lakes from the invasion superhighway using a Welch two-sample t test.

We determined areas of the United States that are at a high-risk for invasions by looking at connections that were from an invaded lake going to an uninvaded lake. We used the mid-point of the receiving lake to assign it to a county in the United States (67) or census geographic units in southern Canada (71). We summed the weights of all connections that terminated in the county and summed the weights of all connections that were terminated at an uninvaded lake in which the origin lake was invaded by one or both *Dreissena* and *Myriophyllum*. We then calculated the percentage of weighted incoming connections that were at high-risk (invaded to uninvaded) for each county.

## Supplementary Material

pgac075_Supplemental_File

## Data Availability

The raw spatiotemporal data for this project were generated by users of the Fishbrain app and obtained through a data-sharing agreement. Requests for raw data should be directed to Johnan Attby of Fishbrain at johan@fishbrain.com. Derived data supporting the findings of this study and interactive maps are openly available and hosted on Zenodo at https://doi.org/10.5281/zenodo.6388121.
