# Correction to: Big data from a popular app reveals that fishing creates superhighways for aquatic invaders

**DOI:** 10.1093/pnasnexus/pgad461

**Published:** 2024-01-30

This is a correction to: Jessica L Weir, Kirsten Vacura, Jay Bagga, Adam Berland, Kieran Hyder, Christian Skov, Johan Attby, Paul A Venturelli, Big data from a popular app reveals that fishing creates superhighways for aquatic invaders, *PNAS Nexus*, Volume 1, Issue 3, July 2022, pgac075, https://doi.org/10.1093/pnasnexus/pgac075

During a retroactive audit conducted by *PNAS Nexus*, it was discovered that this paper was missing a statement acknowledging compliance with the *PNAS Nexus* Human and Animal Participants and Clinical Trials policy:

Data were collected by the Fishbrain app under the EU's General Data Protection Regulation (GDPR). Third-party sharing is permitted under Fishbrain's terms of service, but the company does not share any personal information about their users. We receive information about each fish catch (only location and time are directly relevant to this study), but the users who logged these fish are only identifiable via a unique numeric code. For this study, we used the lake visitation behavior of individual users in aggregate to infer human-mediated lake linkages across the US. Ball State's institutional review board reviewed this information and granted an exemption.

This error has been corrected in the original article.

