# Supplementary material for: Big data from a popular app reveals that fishing creates superhighways for aquatic invaders
Source: PNAS Nexus. 2022 Jun 8;1(3):pgac075. doi: 10.1093/pnasnexus/pgac075 (PMC9896924; doi:10.1093/pnasnexus/pgac075)
Supplement: pgac075_Supplemental_File [file pgac075_supplemental_file.docx]

**Supplemental Materials for**

Big data from a popular app reveals that fishing creates superhighways for aquatic invaders

Jessica Weir^1*^, Kirsten Vacura^1^, Jay Bagga^2^, Adam Berland^3^, Kieran Hyder^4,5^, Christian Skov^6^, Johan Attby^7^, Paul Venturelli^1^

^1^Department of Biology, Ball State University; Muncie, Indiana, United States.

^2^Department of Computer Science, Ball State University; Muncie, Indiana, United States.

^3^Department of Geography, Ball State University; Muncie, Indiana, United States.

^4^Center for Environment, Fisheries and Aquaculture Science (Cefas); Lowestoft, Suffolk, United Kingdom.

^5^School of Environmental Sciences, University of East Anglia; Norwich, Norfolk, United Kingdom.

^6^Technical University of Denmark; Kongens Lyngby, Denmark.

^7^Fishbrain; Stockholm, Sweden.

*Corresponding author: Jessica Weir

**Email:**  [jit.weir](mailto:xxxxx@xxxx.xxx)@gmail.com

**Author Contributions:**

**Competing Interest Statement:** We disclose the financial interests of Johan Attby, Fishbrain CEO. He provided essential data for analysis and input for revisions but has not influenced the content of the paper to benefit Fishbrain, nor undermined the objectivity of the research. The remaining authors have no competing interests.

**Classification:** Biological Sciences, Ecology

**Keywords:** Invasive Species, Human dimensions, Network, Big Data

**This DOCX file includes:**

Supplemental Text

Supplemental Figures

Supplemental Tables

**Supplemental Text**

Log-logistic Curve

We surveyed the literature to obtain time to mortality data for 20 aquatic invasive species and life stages in North America (Supplementary Table 1). We then fit a log-logistic function to the proportion of species or life stages alive after up to 99 days (Supplementary Figure 1). The predicted proportion of life stages that were alive after a given number of days (0.0-1.0) was used as a multiplier that weighted each connection in terms of transfer risk. This approach assumed that the probability of survival was ~0 after 99 days, which may not be the case for desiccation-resistant life stages such as seeds.

Network and Lake Vulnerability

We demonstrated the utility of the information gained from our methods by identifying lakes that should be prioritized for prevention of spreading aquatic invaders. Highly connected lakes were identified by state for the benefit of local management. Hubs (i.e., highly connected nodes) are lakes that exhibited the greatest connectivity to other lakes in the angler network. Two hubs were identified per state focused separately on *Myriophyllum* and *Dreissena* (Supplemental Table 2) species. Invaded hubs were identified as the lake with the highest weighted connectivity for which the invasive species was present, and uninvaded hubs were identified as the highest degree lakes in each state for which there was no recorded incidence of the invasive species according to national distribution data.

**Supplemental References from Table S1**

1. D. K. Branstrator, L. J. Shannon, M. E. Brown, M. T. Kitson, Effects of chemical and physical conditions on hatching success of Bythotrephes longimanus resting eggs. *Limnol. Oceanogr.* **58**, 2171–2184 (2013).

2. L. Bruckerhoff, J. E. Havel, S. Knight, Survival of invasive aquatic plants after air exposure and implications for dispersal by recreational boats. *Hydrobiologia* **746**, 113–121 (2015).

3. Á. Alonso, P. Castro-díez, NeoBiota Tolerance to air exposure of the New Zealand mudsnail Potamopyrgus antipodarum ( Hydrobiidae , Mollusca ) as a prerequisite to survival in overland translocations. **74**, 67–74 (2012).

4. A. Ricciardi, F. G. Whoriskey, J. B. Rasmussen, Predicting the intensity and impact of Dreissena infestation on native unionid bivalves from Dreissena field density. *Can. J. Fish. Aquat. Sci.* **52**, 1449–1461 (1995).

5. W. J. Choi, S. Gerstenberger, R. F. McMahon, W. H. Wong, Estimating survival rates of quagga mussel (Dreissena rostriformis bugensis) veliger larvae under summer and autumn temperature regimes in residual water of trailered watercraft at Lake Mead, USA. *Manag. Biol. Invasions* **4**, 61–69 (2013).

6. J. E. Havel, L. A. Bruckerhoff, M. A. Funkhouser, A. R. Gemberling, Resistance to desiccation in aquatic invasive snails and implications for their overland dispersal. *Hydrobiologia* **741**, 89–100 (2014).

7. P. L. Fuller, A. J. Benson, G. Nunez, A. Fusaro, M. Nielson, “Channa argus (Cantor, 1842)” (2021).

8. E. A. Darbyson, J. M. Hanson, A. Locke, J. H. M. Willison, Survival of European green crab (Carcinus Maenus L.) exposed to simulated overland and boating-vector transport conditions. *J. Shellfish Res.* **28**, 377–382 (2009).

9. J. E. Havel, Survival of the exotic Chinese mystery snail (Cipangopaludina chinensis malleata) during air exposure and implications for overland dispersal by boats. *Hydrobiologia* **668**, 195–202 (2011).

10. P. C. Darby, R. E. Bennetts, H. F. Percival, Dry down impacts on apple snail (Pomacea paludosa) demography: Implications for wetland water management. *Wetlands* **28**, 204–214 (2008).

**Supplemental Figures and Tables**


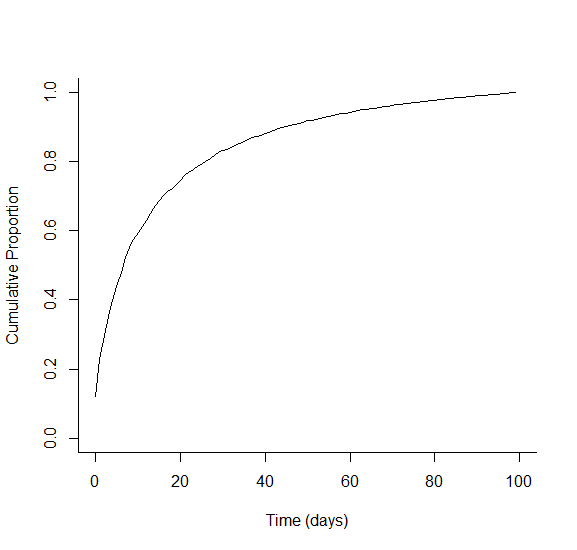


**Figure S1.** The cumulative proportion of time in days between paired lake visits.


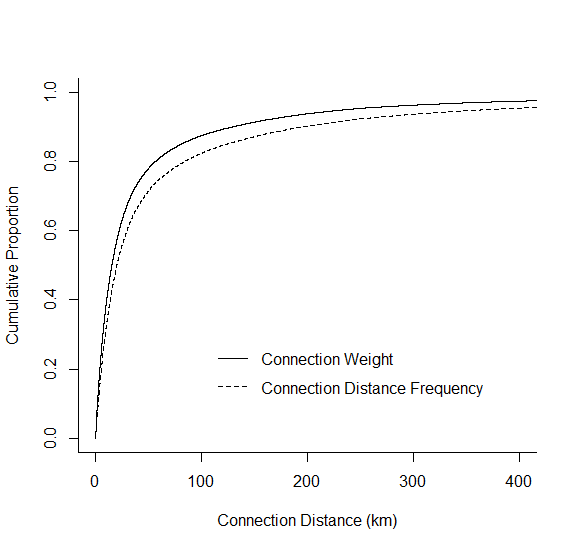


**Figure S2.** The cumulative proportion of total edge connection weight and frequency with the increasing distance for the linear connection between paired lakes (km). Connection weights were calculated as the summed activity of time-weighted movement. Connection distances occurred at up to 4,450 km (not shown).


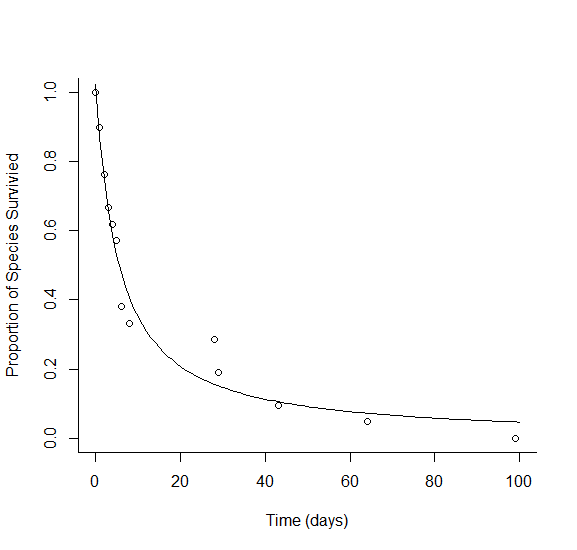


**Figure S3.** Log-logistic function (line) showing the weight assigned to lake connections, representing the proportion of life stages of various aquatic species that are expected to be viable given the number of days between paired lake visits by a single angler. Connections occurring >99 days apart were considered null.

**Table S1.** A list of the primary hub lakes invaded by one or both *Myriophyllum* and *Dreissena* species for each state in the contiguous United States (not including the Great Lakes, which are nationally ranked hubs). Hub lakes are highly connected lakes in any state (i.e., many high-weighted incoming and outgoing edge connections). States are listed in descending ordered according to the degree for the invaded hub lake (i.e., Watts Bar Lake has the greatest connection among all *Myriophyllum* invaded lakes in the US). Uninvaded hubs are not invaded according to national distribution data for the species. States which are not known to have any invasions are indicated with “None”.

| ***Myriophyllum*** | | |
| --- | --- | --- |
| **State** | **Invaded Hub** | **Uninvaded Hub** |
| Tennessee | Watts Bar Lake (35.847°, -84.552°) | J. Percy Priest Reservoir (36.066°, -86.526°) |
| Alabama | Guntersville Lake (34.552°, -86.108°) | Logan Martin Lake (33.443°, -86.264°) |
| Michigan | Kent Lake (42.534°, -83.636°) | Belleville Lake (42.209°, -83.517°) |
| Texas | Lake Travis (30.44°, -98.065°) | Lake Lewisville (33.084°, -96.999°) |
| Massachusetts | Wachusett Reservoir (42.373°, -71.741°) | Spy Pond (42.409°, -71.152°) |
| Kentucky | Kentucky Lake (36.811°, -88.185°) | Taylorsville Lake (38.008°, -85.263°) |
| Vermont | Lake Champlain (44.265°, -73.274°) | Moore Reservoir (44.348°, -71.836°) |
| Illinois | South Pool (42.02°, -88.012°) | Vulcan Lakes (42.216°, -88.301°) |
| Minnesota | Lake Minnetonka (44.942°, -93.55°) | Lake of the Woods (49.076°, -95.005°) |
| Missouri | Lake of the Ozarks (38.167°, -92.965°) | Table Rock Lake (36.589°, -93.555°) |
| Florida | Lake Tohopekaliga (28.214°, -81.397°) | North Community Park Canal (26.301°, -80.265°) |
| Washington | Lake Washington (47.611°, -122.272°) | Sequalitchew Lake (47.112°, -122.601°) |
| New York | Oneida Lake (43.203°, -75.91°) | Little Rainbow Pond (44.359°, -74.325°) |
| Pennsylvania | Lake Nockamixon (40.464°, -75.223°) | Lake Wallenpaupack (41.425°, -75.212°) |
| South Carolina | Lake Wylie (35.025°, -81.015°) | Lake Hartwell (34.593°, -82.88°) |
| Ohio | Nimisila Reservoir (40.93°, -81.524°) | Coe Lake (41.362°, -81.85°) |
| Colorado | Monument Lake (39.09°, -104.881°) | Chatfield Lake (39.552°, -105.065°) |
| California | Lake Berryessa (38.586°, -122.227°) | Puddingstone Reservoir (34.087°, -117.804°) |
| Wisconsin | Lake Winnebago (44.017°, -88.41°) | Lake Delton (43.603°, -89.767°) |
| Utah | Mantua Reservoir (41.505°, -111.933°) | Utah Lake (40.24°, -111.798°) |
| New Jersey | Lake Hopatcong (40.949°, -74.637°) | Newton Lake (39.909°, -75.079°) |
| Connecticut | Lake Candlewood (41.463°, -73.441°) | Maltby Lakes North (41.308°, -72.979°) |
| Rhode Island | Olney Pond (41.891°, -71.432°) | Stillwater Reservoir (41.91°, -71.549°) |
| Virginia | Swift Creek Reservoir (37.44°, -77.689°) | Burke Lake (38.763°, -77.299°) |
| Kansas | Lake Shawnee (39.004°, -95.628°) | Milford Lake (39.201°, -96.988°) |
| Idaho | Lake Pend Oreille (48.149°, -116.395°) | Fernan Lake (47.674°, -116.726°) |
| Oregon | Kirk Pond (44.12°, -123.292°) | Emigrant Lake (42.137°, -122.612°) |
| Louisiana | City Park Pond (29.999°, -90.09°) | Big Lake (29.984°, -90.093°) |
| Arizona | Encanto Lagoon (33.475°, -112.089°) | Lake Pleasant (33.86°, -112.291°) |
| Indiana | Monroe Lake (39.028°, -86.467°) | Wolf Lake (41.673°, -87.518°) |
| Nebraska | Wildwood Lake (41.037°, -96.84°) | Holmes Lake (40.782°, -96.633°) |
| Georgia | Bull Sluice Lake (33.974°, -84.376°) | Lake Sidney Lanier (34.25°, -83.905°) |
| Arkansas | Lake Hamilton (34.411°, -93.076°) | Beaver Lake (36.235°, -93.943°) |
| Maryland | Deep Creek Lake (39.485°, -79.321°) | Loch Raven Reservoir (39.449°, -76.56°) |
| Nevada | Lahontan Reservoir (39.377°, -119.168°) | Lake Mead (36.173°, -114.43°) |
| Maine | Cobbosseecontee Lake (44.243°, -69.952°) | Sebago Lake (43.872°, -70.591°) |
| Oklahoma | Elmer Thomas Lake (34.726°, -98.522°) | Lake Texoma (33.883°, -96.695°) |
| Delaware | Mud Millpond (39.071°, -75.74°) | Silver Lake (39.176°, -75.531°) |
| Mississippi | Ross R Barnett Reservoir (32.433°, -90.034°) | Maywood Lake (34.971°, -89.851°) |
| Iowa | Prairie Lakes (42.478°, -92.458°) | West Okoboji Lake (43.377°, -95.155°) |
| South Dakota | Lake Francis Case (43.441°, -99.244°) | Sheridan Lake (43.972°, -103.478°) |
| North Carolina | Lake Raleigh (35.765°, -78.682°) | Lake Norman (35.451°, -80.963°) |
| New Mexico | Cochiti Lake (35.623°, -106.312°) | Tingley Beach (35.087°, -106.675°) |
| New Hampshire | Mascoma Lake (43.627°, -72.147°) | Lake Winnipesaukee (43.601°, -71.341°) |
| Montana | Canyon Ferry Lake (46.435°, -111.543°) | Flathead Lake (47.884°, -114.099°) |
| West Virginia | Mud River Lake (38.148°, -82.058°) | Skin Creek Lake (38.977°, -80.45°) |
| North Dakota | None | Lake Sakakawea (47.787°, -102.333°) |
| Wyoming | None | West Tensleep Lake (44.261°, -107.216°) |
| ***Dreissena*** | | |
| **State** | **Invaded Hub** | **Uninvaded Hub** |
| Texas | Lake Lewisville (33.084°, -96.999°) | Phillips North Lake (33.141°, -96.886°) |
| Tennessee | Watts Bar Lake (35.847°, -84.552°) | J. Percy Priest Reservoir (36.066°, -86.526°) |
| Alabama | Guntersville Lake (34.552°, -86.108°) | Logan Martin Lake (33.443°, -86.264°) |
| Michigan | Kent Lake (42.534°, -83.636°) | Belleville Lake (42.209°, -83.517°) |
| Kentucky | Kentucky Lake (36.811°, -88.185°) | Taylorsville Lake (38.008°, -85.263°) |
| Vermont | Lake Champlain (44.265°, -73.274°) | Lake Paran (42.933°, -73.233°) |
| Minnesota | Lake Minnetonka (44.942°, -93.55°) | Cedar Lake (44.96°, -93.321°) |
| Missouri | Lake of the Ozarks (38.167°, -92.965°) | Table Rock Lake (36.589°, -93.555°) |
| Oklahoma | Lake Texoma (33.883°, -96.695°) | Stanley Draper Lake (35.35°, -97.356°) |
| Colorado | Pueblo Reservoir (38.268°, -104.751°) | Chatfield Lake (39.552°, -105.065°) |
| Nevada | Lake Mead (36.173°, -114.43°) | Sparks Marina (39.533°, -119.729°) |
| Washington | Lake Washington (47.611°, -122.272°) | Long Lake (47.831°, -117.763°) |
| Indiana | Wolf Lake (41.673°, -87.518°) | Unnamed Lake (40.716°, -85.286°) |
| New York | Oneida Lake (43.203°, -75.91°) | Little Rainbow Pond (44.359°, -74.325°) |
| Illinois | Nippersink Lake (42.414°, -88.144°) | South Pool (42.02°, -88.012°) |
| Utah | Deer Creek Reservoir (40.443°, -111.493°) | Utah Lake (40.24°, -111.798°) |
| Ohio | Nimisila Reservoir (40.93°, -81.524°) | Coe Lake (41.362°, -81.85°) |
| California | Dixon Reservoir (33.16°, -117.044°) | Puddingstone Reservoir (34.087°, -117.804°) |
| Kansas | Milford Lake (39.201°, -96.988°) | Southlake Pond (38.926°, -94.775°) |
| Wisconsin | Lake Winnebago (44.017°, -88.41°) | Minocqua Lake (45.872°, -89.692°) |
| Arizona | Lake Pleasant (33.86°, -112.291°) | Chaparral Lake (33.512°, -111.908°) |
| Connecticut | Lake Candlewood (41.463°, -73.441°) | Maltby Lakes North (41.308°, -72.979°) |
| Iowa | West Okoboji Lake (43.377°, -95.155°) | Lake McBride (41.795°, -91.531°) |
| Nebraska | Glenn Cunningham Lake (41.34°, -96.051°) | Holmes Lake (40.782°, -96.633°) |
| Arkansas | Murray Lake (34.867°, -92.464°) | Beaver Lake (36.235°, -93.943°) |
| North Dakota | Devils Lake (48.046°, -98.998°) | Lake Sakakawea (47.787°, -102.333°) |
| Pennsylvania | Conneaut Lake (41.623°, -80.305°) | Lake Nockamixon (40.464°, -75.223°) |
| South Dakota | Lake Francis Case (43.441°, -99.244°) | Sheridan Lake (43.972°, -103.478°) |
| Virginia | Little Creek Reservoir (37.358°, -76.83°) | Burke Lake (38.763°, -77.299°) |
| Massachusetts | Laurel Lake (42.325°, -73.269°) | Wachusett Reservoir (42.373°, -71.741°) |
| Montana | Canyon Ferry Lake (46.435°, -111.543°) | Flathead Lake (47.884°, -114.099°) |
| Maryland | Hydes Quarry (39.565°, -77.073°) | Loch Raven Reservoir (39.449°, -76.56°) |
| Delaware | None | Silver Lake (39.176°, -75.531°) |
| Florida | None | North Community Park Canal (26.301°, -80.265°) |
| Georgia | None | Lake Sidney Lanier (34.25°, -83.905°) |
| Idaho | None | Lake Pend Oreille (48.149°, -116.395°) |
| Louisiana | None | City Park Lake (29.999°, -90.09°) |
| Maine | None | Sebago Lake (43.872°, -70.591°) |
| Mississippi | None | Ross R Barnett Reservoir (32.433°, -90.034°) |
| New Hampshire | None | Lake Winnipesaukee (43.601°, -71.341°) |
| New Jersey | None | Newton Lake (39.909°, -75.079°) |
| New Mexico | None | Tingley Beach (35.087°, -106.675°) |
| North Carolina | None | Lake Norman (35.451°, -80.963°) |
| Oregon | None | Emigrant Lake (42.137°, -122.612°) |
| Rhode Island | None | Stillwater Reservoir (41.91°, -71.549°) |
| South Carolina | None | Lake Wylie (35.025°, -81.015°) |
| West Virginia | None | Skin Creek Lake (38.977°, -80.45°) |
| Wyoming | None | West Tensleep Lake (44.261°, -107.216°) |

**Table S2.** A summary of literature review for details on the overland transport survivability of 20 aquatic invasive species or life stages in North America.

| **Species** | **Life Stage** | **Dispersal Method** | **Time to Mortality (Days)** |
| --- | --- | --- | --- |
| Spiny waterflea *(Bythotrephes longimanus)* (1) | Resting Egg | Overland transport (drying) | 0 |
| Brook trout *(Salvelinus fontinalis)* | Adult | Bucket without water | 0 |
| Watermilfoil *(Myriophyllum spicatum)* (2) | Single Stem | Overland transport (drying) | 1 |
| Watermilfoil (2) | Coiling | Overland transport (drying) | 2 |
| Curly-leaf pondweed *(Potamogeton crispus)* (2) | Single Stem | Overland transport (drying) | 1 |
| Curly-leaf pondweed (2) | Turions | Overland transport (drying) | 28 |
| New Zealand Mud Snail *(Potamopyrgus antipodarum)* (3) | N/A | Overland transport | 2 |
| Hydrilla *(Hydrilla verticillata)* (2) | N/A | Aerial exposure | 2 |
| Bait fishes | Adult | Bait buckets | 4 |
| Zebra mussel *(Dreissena polymorpha)* (4) | Adult | Small, trailered boats | 5 |
| Quagga mussel *(Dreissena bugensis)* (5) | Veliger | Boats | 27 |
| Quagga mussel (4) | Adult | Small, trailered boats | 5 |
| Banded mystery snail *(Viviparous georgianus)* (6) | Juvenile | Overland transport | 5 |
| Banded mystery snail (6) | Adult | Overland transport | 42 |
| Snakehead *(Channa argus)* (7) | Adult | Overland transport | 5 |
| Green Crab *(Carcinus maenas)* (8) | Juvenile | Fishing gear and boats | 7 |
| Chinese mystery snail *(Cipangopaludina chinensis)* (9) | Juvenile | Small, trailered boats | 28 |
| Chinese mystery snail (6) | Adult | Overland transport | 63 |
| Mud bithynia/faucet snail *(Bithymia tentaculata)* (6) | Adult | Overland transport | 15 |
| Florida apple snail *(Pomacea paludosa)* (10) | N/A | N/A | 98 |
